# IspH–RPS1 and IspH–UbiA: “Rosetta stone” proteins†
†Electronic supplementary information (ESI) available: Zoomed-in view of IspH–RPS1 network, UbiA superfamily, LC-MS analysis of IspH–RPS1 catalysis, simulation of EPR spectrum, and IspH–RPS1/Rho interaction. See DOI: 10.1039/c5sc02600h



**DOI:** 10.1039/c5sc02600h

**Published:** 2015-09-07

**Authors:** Guodong Rao, Bing O'Dowd, Jikun Li, Ke Wang, Eric Oldfield

**Affiliations:** a Department of Chemistry , University of Illinois at Urbana – Champaign , Urbana , IL 61801 , USA . Email: eoldfiel@illinois.edu

## Abstract

The protein IspH, (*E*)-1-hydroxy-2-methyl-but-2-enyl 4-diphosphate (HMPPP) reductase, is an essential 4Fe–4S cluster-containing protein in the methylerythritol phosphate pathway for isoprenoid biosynthesis. Using a sequence similarity network we found that there are >400 IspH proteins that are about twice as large as most of the IspHs studied to date since their IspH domains are fused to either the ribosomal protein S1 (RPS1), or to a UbiA (4-hydroxybenzoate octaprenyltransferase)-like protein. Many of the IspH–RPS1 proteins are present in anaerobes found in the human gut and some, such as *Clostridium botulinum*, *C. tetani* and *Fusobacterium nucleatum*, are pathogens. The IspH–UbiAs are all found in sulfate-reducing anaerobes. The IspH domains in IspH–RPS1 are fused to 4 and in a few cases 6 tandem repeats in RPS1 that, in most organisms, bind to mRNA or form part of the bacterial ribosome. Mutants in which the four RPS1 domains were sequentially eliminated had similar IspH activity as wild-type protein, indicating they are not essential for IspH catalysis. Overall, the results are of interest since they represent the first isolation of a catalytically active IspH–RPS1, as well as the identification of IspH–UbiA hybrids, two “Rosetta stone” proteins that are likely to be functionally related—IspH producing the isoprenoids required for a UbiA-like prenyltransferase; the IspH–RPS1 hybrids, perhaps, being involved in the stringent response or as Fe/O_2_ sensors.

## Abstract 2

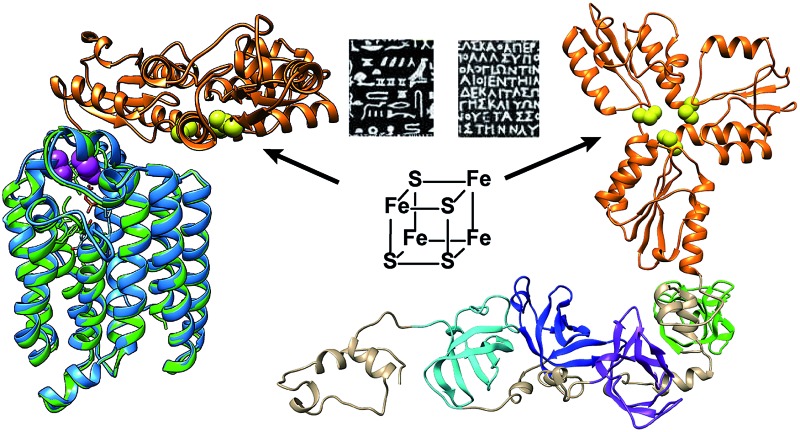
IspH forms fusion hybrids with RPS1 as well as UbiA, examples of Rosetta stone proteins.

## Introduction

**Scheme 1:**
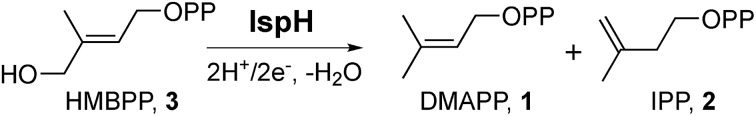
IspH catalysis. The reaction involves the 2H^+^/2e^–^ reductive dehydroxylation of (*E*)-1-hydroxy-2-methylbut-2-enyl-4-diphosphate (**3**) to dimethylallyl diphosphate (**1**) and isopentenyl diphosphate (**2**).

The methylerythritol phosphate pathway is responsible for the production of the terpene building blocks dimethylallyl diphosphate (DMAPP, **1**, Scheme 1) and isopentenyl diphosphate (IPP, **2**) in most bacteria, apicomplexan parasites, and plants. Its component enzymes are all essential for survival, so there is interest in their inhibition^1,2^ for the development of drugs to treat infectious diseases, such as malaria and tuberculosis, with fosmidomycin,^3^ which inhibits deoxyxylulose 4-phosphate reductoisomerase, having reached clinical trials for malaria.^4^ In addition, targeting the pathway could be of use for herbicide development. IspH, (*E*)-1-hydroxy-2-methyl-but-2-enyl 4-diphosphate (HMBPP, **3**) reductase, also known as LytB (but not to be confused with the *N*-acetylglucosaminidase LytB from *Streptococcus* spp.), is the last enzyme in the pathway.^5^ IspH is a 4Fe–4S cluster-containing protein^6^ that catalyzes the 2H^+^/2e^–^ reductive dehydroxylation of HMBPP into a ∼1 : 5 mixture of dimethylallyl diphosphate and isopentyl diphosphate. Its structure and mechanism of action have been studied extensively, both experimentally^7–13^ and computationally,^14–16^ and several inhibitors have been developed.^7^ The mechanism of action is remarkable and the consensus view now is that IspH utilizes a bio-organometallic mechanism in which an allyl species binds to the Fe–S cluster.^14–16^

Despite their now well-known role in isoprenoid biosynthesis, IspHs (known as LytBs at the time) from *E. coli* and *Campylobacter jejuni* were first identified and investigated almost 20 years ago in studies of the so-called “stringent response”, in bacteria.^17,18^ The stringent response is a stress response that can arise from diverse insults and is aimed at limiting growth to promote survival. An IspH mutant *E. coli* strain WV7 (ref. 18) induced the stringent response and exhibited a penicillin-tolerant phenotype at restrictive temperatures (42 °C). This phenotype could be complemented by wild-type *E. coli* IspH or *C. jejuni* IspH,^17^ but not by a *C. jejuni* IspH Q265H mutant and it was proposed that the wild-type IspH could interact “directly or indirectly” with RelA.^18^ RelA is an enzyme that has been shown to bind to the bacterial ribosome^19^ and is responsible for the biosynthesis of the alarmone, (p)ppGpp, guanosine tetraphosphate (or pentaphosphate), the key regulator involved in the bacterial stringent response.^20^ In addition, RelA is an emerging central regulator of multidrug tolerance and persistence.^21^ If the interaction between IspH and RelA were disrupted, the stringent response would be induced.

In other work it has been noted that some bacterial proteins appear to contain a fusion in which IspH is linked to another ribosomal protein, RPS1, the ribosomal protein small (30S ribosome) protein 1 (ref. 22 and 23). Fusion hybrids are often found when two individual proteins have some related activity and are called “Rosetta stone” proteins^24,25^ and RPS1–IspH is given as one example,^25^ although the functional relatedness of the 2 domains has not been proposed. Here, we report initial cloning, expression, purification, activity, inhibition, mutagenesis and spectroscopic results on one IspH–RPS1 protein. In addition, we report that several other proteins form fusion hybrids with IspH, one of which appears to be a functionally related prenyl synthase.

## Materials and methods

### Sequence similarity network

The sequence similarity network for the IspH family proteins (InterPro number IPR003451) was generated by using the Enzyme Function Initiative Enzyme Similarity Tool (EFI-EST, http://efi.igb.illinois.edu/efi-est/). An expectation-value of 10^–120^ was used to construct the network, which was then visualized by using Cytoscape 3.2.1. A single node represents sequences with at least 90% identity, while each edge joins sequences that share an *e*-value of 10^–120^ or smaller.

### Cloning, protein expression, and purification

*CthispH–rps1* and its mutants were amplified from the genomic DNA of *Clostridium thermocellum* strain VPI 7372 [ATCC® 27405™] by polymerase chain reaction. The amplification product was digested with SacI-HF and SalI-HF (New England Biolabs, MA) and cloned into the pET-28a (+) vector (Novagen, WI). The plasmid with the correct insert was transformed into *E. coli* BL21-CodonPlus (DE3)-RIPL competent cells (Agilent, CA). For protein expression, six liters of LB broth supplemented with 25 mg mL^–1^ kanamycin and 17 mg mL^–1^ chloramphenicol were inoculated with a 0.5% overnight culture and grown at 37 °C to an O.D._600_ of 0.6–0.8. Protein expression was induced by addition of isopropyl β-d-1-thiogalactopyranoside to a final concentration of 1 mM and the cultures were grown at 28 °C for a further 24 h. The cells were then harvested and stored at –80 °C until further use. CthIspH–RPS1 and all mutants were purified by using column chromatography employing a Ni-NTA Hispur™ resin (Fisher, NY), according to the manufacturer's instructions. Briefly, cell pellets were thawed and suspended in the loading buffer (5 mM imidazole, 50 mM Tris/HCl, 150 mM NaCl, pH = 8.0) containing a protease inhibitor cocktail tablet (Roche) and lysed by sonication. The cell debris was discarded after centrifugation and the clear cell lysate was loaded onto the resin. The resin was then washed (50 mM imidazole, 50 mM Tris/HCl, 150 mM NaCl, pH = 8.0) and the desired protein eluted (300 mM imidazole, 50 mM Tris/HCl, 150 mM NaCl, pH = 8.0). Imidazole was removed by dialysis against storage buffer (50 mM Tris/HCl, 150 mM NaCl, 1 mM DTT, 5% glycerol, pH = 8.0). The molecular weight of wild-type CthIspH–RPS1 was confirmed with MALDI-TOF (Bruker UltrafleXtreme, Boston, MA) mass spectrometry. Purity of the proteins was checked with SDS-PAGE. Protein concentrations were determined by using the Bradford assay. The concentrations of the [Fe_4_S_4_]^2+^ clusters were measured by using UV-Vis spectroscopy. The extinction coefficient at 410 nm was taken to be 15 000 M^–1^ cm^–1^.^26^

### Fe–S cluster reconstitution

The Fe_4_S_4_ cluster of CthIspH–RPS1 and its mutants was reconstituted by using purified *Azotobacter vinelandii* IscS protein. AvIscS was expressed from a plasmid which was the kind gift from Professor James A. Imlay. Typically, as-purified IspH–RPS1 was concentrated to ∼2 mL (∼50–100 μM), degassed by bubbling nitrogen through the solution, transferred into an anaerobic chamber (Coy Labs, Grass Lake, IL), then stirred gently in the chamber overnight in order to equilibrate with the inert atmosphere. To the gently stirred protein solution was added 0.5 mM Fe(NH_4_)_2_(SO_4_)_2_, 2.5 mM l-cysteine, 5 mM DTT and ∼0.1 μM IscS. The reaction was incubated for several hours until Fe_4_S_4_ incorporation was satisfactory, taken to be an *A*_410_/*A*_280_ ratio of ∼0.4. The resulting protein solution was then centrifuged to remove any precipitate, desalted by using a PD10 column (Agilent, CA) and concentrated as necessary. IscS was not removed from the sample since it represents only ∼0.1% of the total protein.

### Enzyme kinetics and inhibition assay

The CthIspH kinetics and inhibition assays were performed by using the methyl viologen method described previously.^27^ The final assay solution contained 40 nM enzyme, 50 μM HMBPP, 40 μM dithionite and 2 mM methyl viologen. The reactions were monitored at 606 nm. *K*_m_ and *k*_cat_ were fitted by using a Michaelis–Menten relation in Origin 9.1. The extinction coefficient for the methyl viologen radical cation at 606 nm was taken to be 13 700 M^–1^ cm^–1^.^28^

### Electron paramagnetic resonance (EPR) spectroscopy

All sample preparation procedures were carried out in an anaerobic chamber. Reconstituted and desalted CthIspH–RPS1 was concentrated to ∼0.3–0.4 mM and 20 equivalents of Na_2_S_2_O_4_ and the ligand of interest, added. Samples (and no-ligand controls) were incubated for 30 min and then transferred into an EPR sample tube. EPR experiments were performed at X-band (9 GHz) using a Varian E-122 spectrometer together with an Air Products (Allentown, PA) helium cryostat. Spectra were obtained at 10 K at a 1 mW power level.

### Protein fingerprint analysis

The identity of the *E. coli* transcription termination factor Rho was assigned by protein fingerprint analysis in the Protein Science Facility at the University of Illinois at Urbana-Champaign. Briefly, the sample containing the unknown protein was trypsin-digested and the resulting mixture analyzed by using electrospray ionization (ESI) tandem mass spectrometry in positive ion mode. The results were compared against a protein database using the Mascot program.^29^

### Synthetic protocols

All reagents were purchased from Aldrich (Milwaukee, WI). The purity of compounds investigated were confirmed by ^1^H, ^13^C and ^31^P NMR spectroscopy at 400 MHz or 500 MHz performed on Varian (Palo Alto, CA) Unity spectrometers. High-resolution mass spectra were obtained at the University of Illinois Mass Spectrometry Laboratory. Cellulose thin layer chromatography (TLC) plates were visualized by using iodine or a sulphosalicylic acid–ferric chloride stain. The syntheses and characterization of **4–12** were described previously.^30^

### 4-Hydroxybut-2-ynyl-*S*-thiolodiphosphate (**13**)

tris-(Tetra-*n*-butylammonium)thiodiphosphate was prepared using a literature procedure.^31^ 1-Chloro-4-hydroxy-2-butyne (52 mg, 0.5 mmol) in CH_3_CN (0.5 mL) was treated with 1.38 g (1.5 mmol) tris-(tetra-*n*-butylammonium)thiodiphosphate in CH_3_CN (2 mL) at 0 °C. The reaction mixture was allowed to warm to room temperature over 8 h, then solvent was removed under reduced pressure. The residue was dissolved in 1 mL of cation-exchange buffer (49 : 1 (v/v) 25 mM NH_4_HCO_3_/2-propanol) and passed over 90 equiv. of Dowex AG50W-X8 (100–200 mesh, ammonium form) cation-exchange resin, pre-equilibrated with two column volumes of the same buffer. The product was eluted with two column volumes of the same buffer, flash frozen, and lyophilized. The resulting powder was dissolved in 1 mL of 50 mM NH_4_HCO_3_, 2-propanol/CH_3_CN (1 : 1 (v/v), 2 mL) added, and the mixture mixed on a vortex mixer, then centrifuged for 5 min at 2000 rpm. The supernatant was decanted. This procedure was repeated three times and the supernatants were combined. After solvent removal and lyophilization, followed by flash chromatography on a cellulose column (2 : 1 : 1 (v/v/v) 2-propanol/CH_3_CN/50 mM NH_4_HCO_3_), a white solid 24 mg (yield 15%) was obtained. ^1^H NMR (400 MHz, D_2_O): *δ*, 4.05–4.03 (m, 2H), 3.44–3.40 (m, 2H); ^13^C NMR (125.7 MHz, D_2_O): *δ*, 82.46, 81.03, 49.89, 18.43; ^31^P NMR (161.9 MHz, D_2_O): *δ*, 7.83 (d, *J* = 26.4 Hz), –9.50 (d, *J* = 26.4 Hz); high-resolution mass spectrometry (HRMS, ESI) calcd for C_4_H_9_O_7_P_2_S [M + H]^+^, 262.9544; found 262.9543.

## Results and discussion

### IspH–RPS1: a Rosetta stone protein present in many gut bacteria, and some human pathogens

**Fig. 1:**
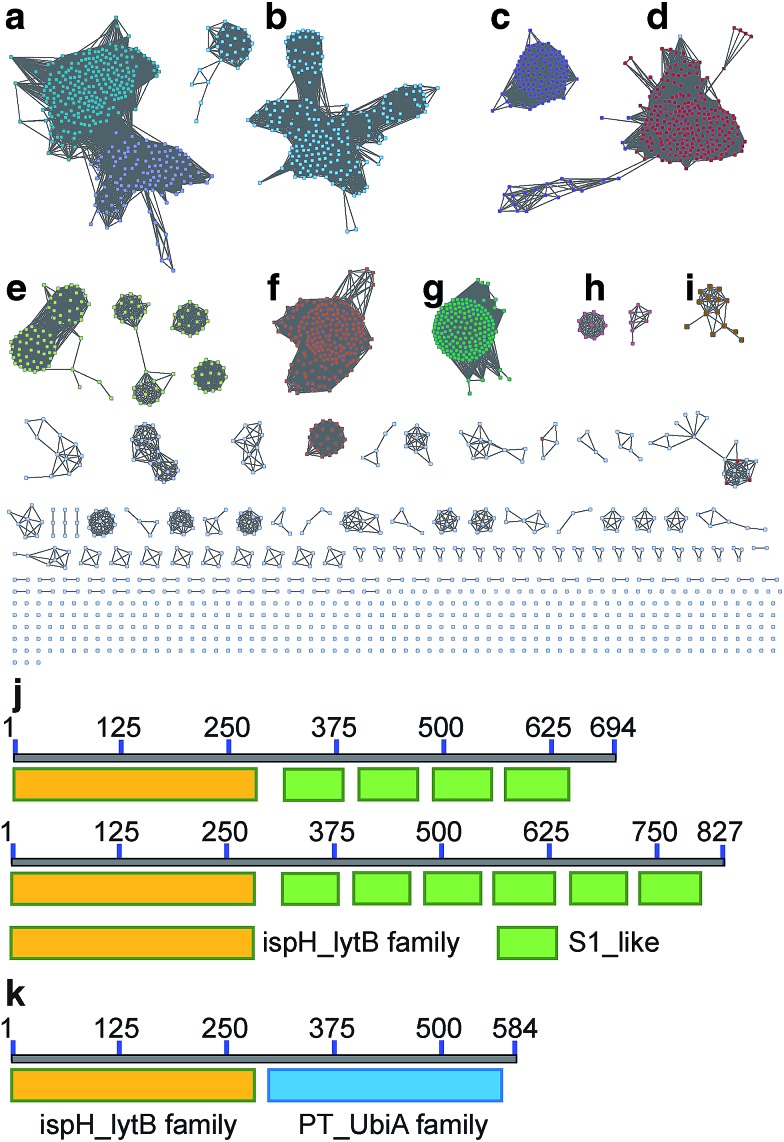
Sequence similarity network of the IspH family proteins with 15 660 members constructed at an expectation-value (*e*-value) of 10^–120^. A single node represents sequences with at least 90% similarity. Major clusters of interest include: (a) gammaproteobacteria (dark cyan, upper left) and betaproteobacteria (blue, lower right); (b) alphaproteobacteria (cyan); (c) firmicutes (purple); (d) firmicutes (with very long sequences, red); (e) bacteroidetes (yellow); (f) actinobacteria (orange); (g) cyanobacteria and plants (green); (h) apicomplexa (pink, genus *Plasmodium*, the malaria parasites) and (i) desulfobacterales (brown, an order of deltaproteobacteria) (j) the architectures of IspH–RPS1 with 4 (top) and 6 (bottom) S1 repeats; (k) the architectures of IspH–UbiA.

We first investigated the network of IspH family proteins by using the Enzyme Similarity Tool from the Enzyme Function Initiative (EFI-EST; http://efi.igb.illinois.edu/efi-est/) to generate a sequence similarity network for IspH family proteins, and visualized the results by using Cytoscape.^32–34^ An overview of 15 660 IspHs is shown in Fig. 1. Most can be sorted into clusters based on the phylum and class of the organism. In a network with an expectation value of 10^–120^, the major clusters shown in Fig. 1 are as follows: (a) gammaproteobacteria (dark cyan, upper left) and betaproteobacteria (blue, lower right); (b) alphaproteobacteria (cyan); (c) firmicutes (purple); (d) firmicutes (with very long sequences, red); (e) bacteroidetes (yellow); (f) actinobacteria (orange); (g) cyanobacteria and plants (green); (h) apicomplexa (pink, genus *Plasmodium*, the malaria parasites) and (i) desulfobacterales (brown, an order of deltaproteobacteria).

What is of particular interest about these bioinformatics results is that, unlike most bacterial IspHs—which typically have ∼300 residues (*e.g. E. coli*, *N* = 316 residues, and *Aquifex aeolicus*, *N* = 297 residues)—the IspHs found in cluster D (and a few isolated nodes) are much larger, with *N* ∼ 600–850 residues. In most cases this is due to a fusion with several ribosomal protein S1-like tandem repeats, found in the ribosomal protein S1 (RPS1) in the 30S subunit of the bacterial ribosome. The basic IspH–RPS1 architectures are shown schematically in Fig. 1j. Notably, essentially all of the organisms that have the IspH–RPS1 fusion are anaerobic bacteria. Among the 447 IspH–RPS1 sequences, 157 are from anaerobes. The other 290 are not annotated, but are primarily from anaerobic species. As shown in the zoomed-in view of the IspH–RPS1 network (ESI, Fig. S1†), the majority of the IspH–RPS1s are from firmicutes, the low G + C Gram-positives, including many members from the genus *Clostridium*. They include pathogens such as *C. tetani* and *C. botulinum* (the causative agents of tetanus and botulism, respectively), as well as the industrially valuable strain, *C. acetobutylicum*. A few IspH–RPS1s are from Gram-negatives and typically contain 6 S1 repeats, Fig. 1j, one example being *Fusobacterium nucleatum*. This organism plays a role in periodontal disease as well as being associated with colorectal carcinoma^35,36^ where it is involved in protecting tumors from immune system (NK cell) attack.^37^ We also found that 37% of the IspH–RPS1s are present in the human gut, examples being *Clostridium* sp. SS2-1, *Eubacterium hallii*, *Subdoligranulum variabile*, *Clostridium leptum*, *Coprococcus eutactus*, *Clostridium* sp. L2-50, *Anaerotruncus colihominis*, and *Clostridium asparagiforme*, the first two organisms being the 7^th^ and 9^th^ most abundant gut bacteria.^38^

As noted in the book chapter by Pellegrini and Graeber,^25^ the IspH–RPS1 fusion hybrids are thought to be examples of Rosetta stone proteins, functionally-related fusion proteins, rather than having arisen from random fusion events. However, there have been no proposals as to what the non-IspH function might be, rather, it is the high occurrence of IspH–RPS1 hybrids that is suggestive of functional relatedness.

### IspH forms other fusion hybrids

**Fig. 2:**
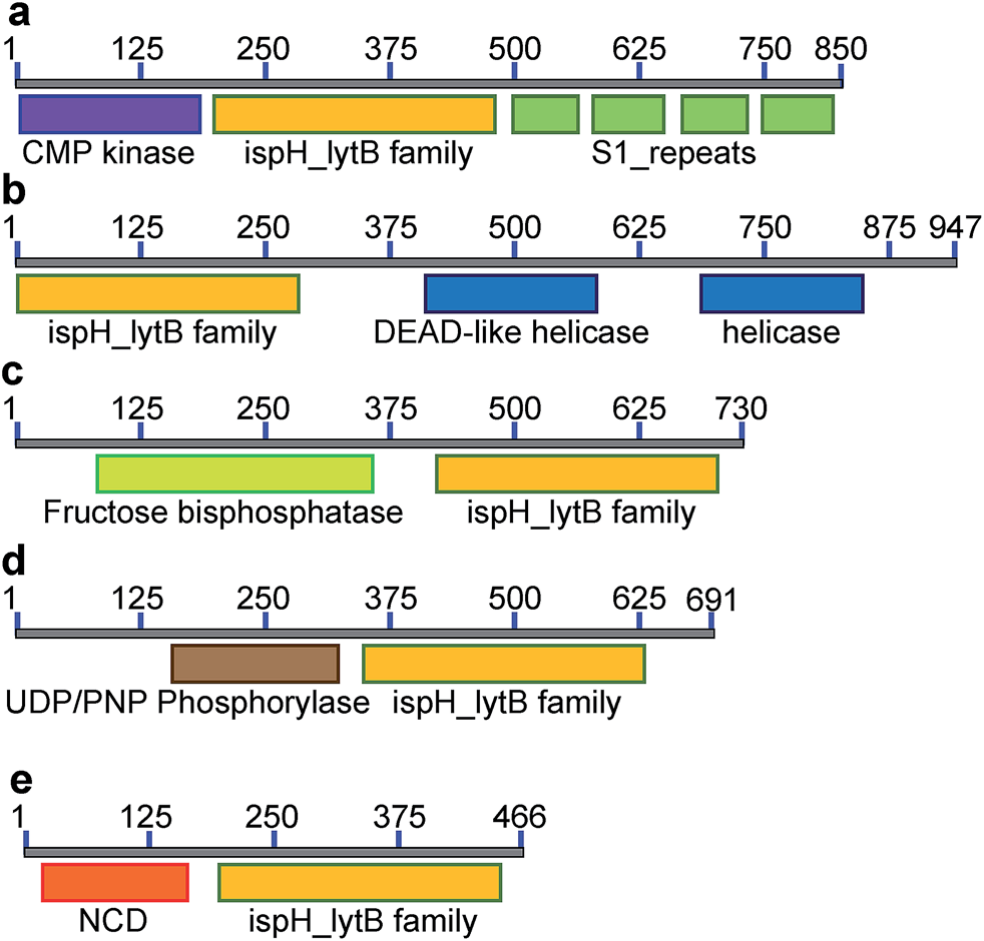
Other IspH fusion proteins found from the sequence similarity network. (a) CMP–IspH–RPS1 from *Candidatus Arthromitus* SFB-*X* (*X* = 1, 2, 3, 4, 5, mouse-NL, and rat). (b) IspH–helicase, from *Bacteroidales bacterium* CF. (c) FBP–IspH from *Chromera velia*. (d) UDP–IspH from *Frankia* sp. (QA3., EUN1f., and strain EAN1pec). (e) IspH with an *N*-terminal conserved domain, from photosynthetic organisms.

Inspired by the observation of large numbers of IspH–RPS1 fusion proteins, we next examined the sequences and conserved domains of all IspHs in the sequence similarity network. In addition to the IspH–RPS1 fusions and the plant/protozoan IspHs (that appear to have an *N*-terminal plastid-localization domain), there are five other types of IspH fusion annotated (Fig. 1k and 2). The most abundant fusion is between IspH and a putative prenyltransferase, typically annotated as a UbiA (4-hydroxybenzoate octaprenyltransferase) family protein. There were 47 such sequences found, primarily in the Gram-negative sulfate-reducing bacteria desulfobacterales (cluster (i) in the Cytoscape network, brown, Fig. 1i) in which the UbiA-like prenyltransferase domain was fused to the *C*-terminus of IspH, Fig. 1k. UbiAs form a superfamily of proteins, Fig. S2,† typically thought to contain ∼9 trans-membrane helices, and the X-ray structure of UbiA has recently been reported.^39^ The function of the UbiA-annotated domain in the IspH–UbiAs, categorized as PT_UbiA_5, is unknown, however, it has been reported that ubiquinones are not detected in sulfate-reducing bacteria;^40^ that menaquinone-7 is the major respiratory quinone in these organisms; and a separate UbiA/MenA is evident in their genomes. It seems likely, nevertheless, that the UbiA domain is involved in prenyltransferase activity, not least since there are two “DXXXD” repeats present, typical of prenyl synthases and transferases, and the fact that IspH appears to be fused to a second prenyltransferase would be of interest since it suggests that the fusion may be functionally significant: a true Rosetta stone protein. In addition, the hybrid protein would likely be membrane-bound. Four other types of fusion were found only in single species (or several strains of the same species): CMP kinase; helicases; fructose bisphosphatase and UDP/PNP phosphorylase (Fig. 2a–d). Plus, in a recent report,^41^ IspHs from a cyanobacterium and a plant, photosynthetic organisms, were proposed to have an extra *N*-terminal conserved domain (NCD) and a conserved Tyr (Fig. 2e), to protect the enzyme from high oxygen levels during photosynthesis, although this domain appears to be a simple IspH modification.

### Organization and structure prediction for IspH–RPS1 and IspH–UbiA

**Fig. 3:**
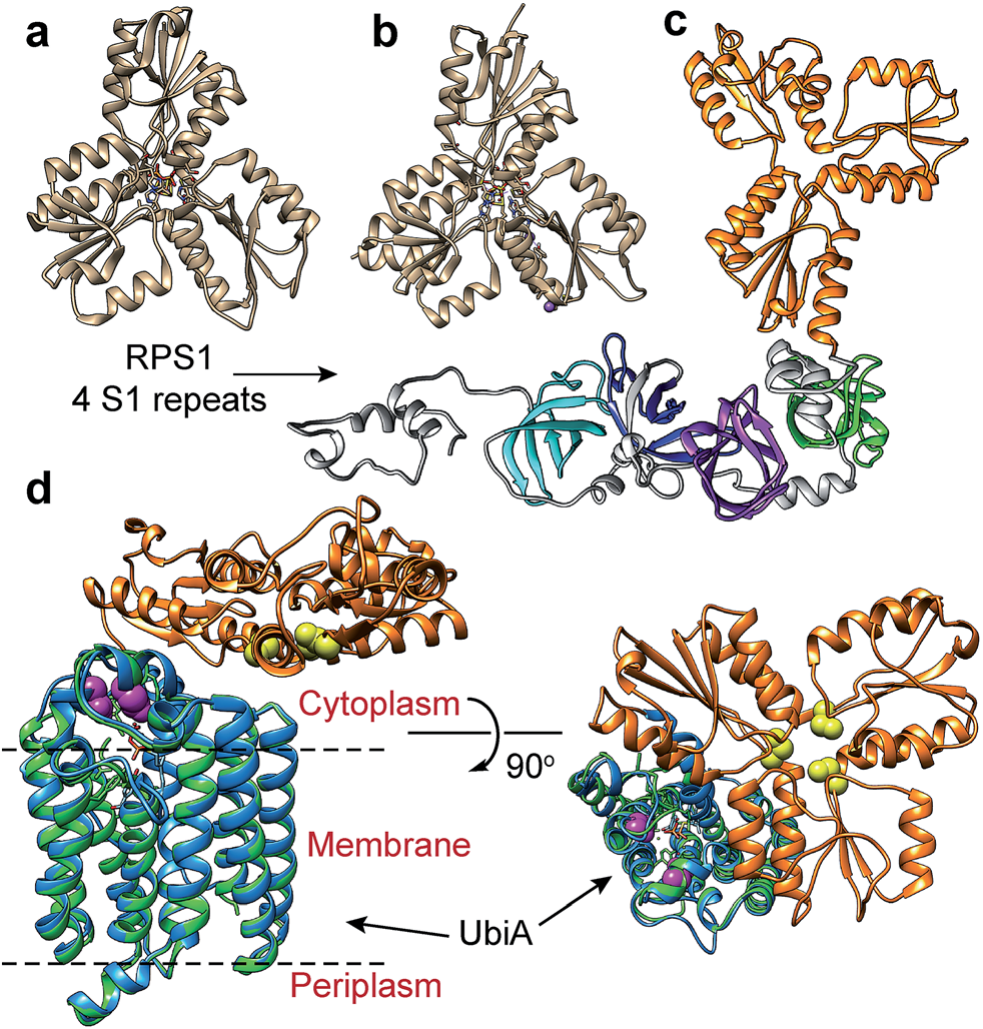
IspH structures and IspH–fusion protein structure predictions. (a) X-ray structure of *E. coli* IspH (PDB ID code: ; 3KE8). (b) X-ray structure of *P. falciparum* IspH (PDB code: ; 4N7B). (c) Phyre2 structure prediction of CthIspH–RPS1. The IspH domains are in orange while the 4 S1 repeats are colored green, purple, blue, or cyan. (d) Phyre2 structure prediction of *Desulfobacula toluolica* IspH–UbiA. Orange: IspH domain. Blue: UbiA domain. Green: X-ray structure of *Aeropyrum pernix* UbiA (PDB ID code: ; 4OD5) yellow spheres: conserved Cys in IspH. Purple spheres: conserved Asp in UbiA domain in IspH–UbiA.

We next sought to begin to investigate the possible structures of the IspH–RPS1 and IspH–UbiA hybrids. RPS1s from most Gram-positive bacteria (*e.g. Bacillus subtilis*) contain 4 S1-like repeats, Fig. 1j (top), while RPS1s from Gram-negatives (*e.g. E*. *coli*) typically contains 6 S1-like repeats, Fig. 1j (bottom). In *E. coli*, the first two repeats are used for ribosome binding,^42^ and the last four repeats for mRNA binding during translation-initiation. There have been no X-ray structures of any IspH–RPS1s reported. The structures of small bacterial and a protozoal IspH have, however, been reported,^8,43^ and by way of example, we show that of the *E. coli* protein in Fig. 3a, together with that of the *P. falciparium* protein (*N* = 318, the apicoplast-targeting domain being absent), in Fig. 3b.^44^

Both the *E. coli* and *P. falciparum* structures are characterized by a trefoil/clover-leaf arrangement of α/β domains with the catalytic 4Fe–4S cluster at the center of each structure, linked to the protein *via* 3 Cys thiols. Substrate, inhibitor or water molecules occupy the remaining coordination sites. By way of comparison, a computational (Phyre2 program^45^) structure prediction of the *Clostridium thermocellum* IspH–RPS1 protein is shown in Fig. 3c. The trefoil-like IspH domain is well defined, plus there are four more (and less) defined RPS1 domains (which are based on the solution NMR structures of individually expressed RPS1 domains).^23^

A Phyre2 structure prediction of the *Desulfobacula toluolica* (a marine, aromatic compound-degrading, sulfate-reducing bacterium) IspH–UbiA is shown in Fig. 3d. The UbiA domain is essentially that seen in the X-ray structure of *Aeropyrum pernix* UbiA^39^ (the template used by the Phyre2 program) and the 3 conserved Cys (yellow spheres) in the IspH domain and the two Asp rich (DXXXD) sites in the UbiA domain (purple spheres) can be readily identified. The UbiA domain is predicted to contain 9 trans-membrane helices, and the IspH domain presumably lies on the cytoplasmatic side of the membrane, as shown in Fig. 3d, although in most predicted structures this localization was variable.

In addition to the 3 essential Cys found in all IspH family proteins, involved in coordinating the 4Fe–4S cluster, IspH–RPS1s contain significantly more Cys than do the more conventional IspHs: 7.4 *versus* 4.8 on average (in the IspH domain), and some strains (*Clostridium* sp. CAG: 678; *Mogibacterium* sp. CM50) contained as many as 14 Cys. The high cysteine content is probably related to the observation that most of the species are anaerobes, since it has been pointed out that cysteine depletion occurs on a proteome-wide scale on moving from anaerobic to aerobic unicellular organisms.^46^ The range of Cys content in the IspHs is, however, much larger than the proteome-wide average.

At present, there is no information as to whether there are important functional consequences of the IspH–RPS1 (or other) fusions. Thus, as a first step toward answering that question, we next sought to express an IspH–RPS1, the most abundant IspH fusion protein, in order to form a basis for future studies of structure and function.

### Cloning, expression, purification and catalytic activity of IspH–RPS1

Since we did not find any other *ispH* or *rps1* genes in the organisms that harbor *ispH–rps1*, it is almost certain that the IspH domain makes IPP/DMAPP, since mevalonate pathway enzymes are absent in the genome. We first sought to see if IspH–RPS1 could be expressed and purified in an active form, catalyzing IPP/DMAPP formation from HMBPP. We attempted expression of IspH–RPS1s from three bacteria: *F. nucleatum*, *C. acetobutylicum*, and *C. thermocellum*. Initial trials with *F. nucleatum* and *C. acetobutylicum* resulted in low expression yields or inactive protein, although UV-Vis spectra clearly indicated the presence of a Fe–S cluster with *C. acetobutylicum* IspH–RPS1. Fortunately, active (in an HMBPP reduction assay) *C. thermocellum* (Cth) IspH–RPS1 could be readily expressed in *E. coli* with moderate yields (∼3 mg L^–1^). *C. thermocellum* is an anaerobic, thermophilic bacterium that is of commercial interest since it catalyzes the direct conversion of crystalline cellulosic biomass into ethanol.^47^

**Fig. 4:**
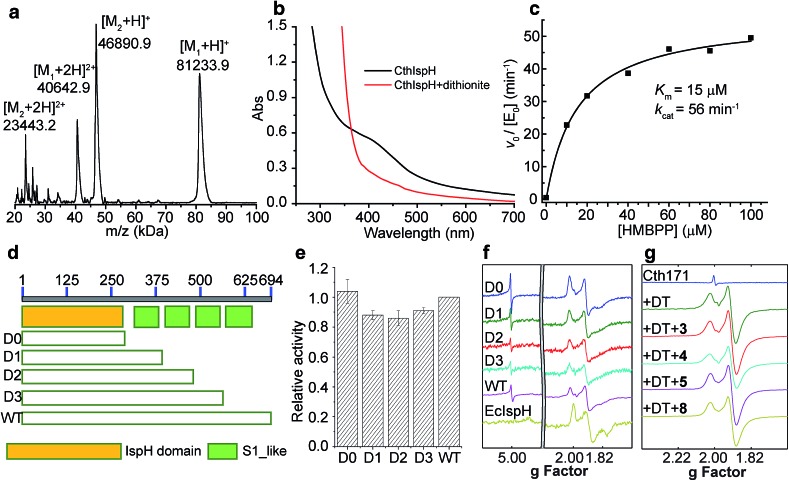
Characterization of CthIspH–RPS1 and its truncation mutants. (a) MALDI-TOF spectrum of purified CthIspH. M_1_: CthIspH–RPS1; M_2_: EcRho, see text for more details. (b) UV-Vis spectrum of CthIspH–RPS1 after iron–sulfur cluster reconstitution (black) and after dithionite reduction (red). (c) Michaelis–Menten kinetics of CthIspH–RPS1. (d) Cartoon of the truncation proteins. D0: no S1 repeat, D1: one S1 repeat, D2: two S1 repeats, D3: three S1 repeats. WT has the four S1 repeats. (e) Relative activities of truncation mutants compared to wild-type protein. Error bars are from *n* = 3 replicates. (f) EPR of reduced CthIspH–RPS1, its truncation mutants, and *E. coli* IspH. (g) EPR of CthIspH–RPS1_1-171, reduced protein, with substrate HMBPP (1) and several ligands. The small peak at *g* = 2.0 in the oxidized protein sample (blue) is due to the small amount of [Fe_3_S_4_]^+^.

*CthispH–rps1* encodes a single polypeptide of 694 residues and contains 4 S1-like repeats in the RPS1 domain. It was purified as an 81.2 kDa protein (with an *N*-terminus His_6_-tag) and its identity was verified by MALDI-TOF with an error of <0.1% (Fig. 4a, [M_1_ + H]^+^, expected: 81309.0, found: 81233.9). However, there was poor iron–sulfur cluster incorporation, typically ∼10%, even when co-expressed with the IscS protein^48^ that catalyzes cluster formation. We thus next used an *in vitro* reconstitution method with an expressed and purified IscS. The UV-Vis absorption spectrum of the reconstituted CthIspH–RPS1 protein at 410 nm, Fig. 4b, is consistent with the presence of an “oxidized” [Fe_4_S_4_]^2+^ cluster. This could be reduced by dithionite to the reduced [Fe_4_S_4_]^+^ cluster, as found with other IspH proteins.^49^

We then examined the catalytic activity of CthIspH–RPS1 in the conversion of HMBPP into DMAPP and IPP by using the methyl viologen assay described previously.^27^ As shown in Fig. 4c, the enzyme has a *k*_cat_ of 56 min^–1^ and a *K*_m_ of 15 μM, at room temperature. The *k*_cat_ is very close to the value found with IspH from the thermophile *Aquifex aeolicus* while the *K*_m_ is 2-fold larger (*k*_cat_ = 1.0 s^–1^; *K*_m_ = 7 μM).^7^ Substrate consumption and product formation were also confirmed by LC-MS (Fig. S3†) and NMR analysis. So, an IspH–RPS1 can be expressed from a thermophile and its activity is similar to that of IspH from another, “IspH-only”, thermophile.

### Truncation mutants: effects on activity and on 4Fe–4S cluster-binding

In order to find out whether the presence of the RPS1 domain had any effects on the catalytic activity of CthIspH–RPS1, we made truncation mutants containing 0, 1, 2 or 3 S1-like repeats (called here D0, D1, D2, D3; cartoon in Fig. 4d). As shown in Fig. 4e, these truncation mutants had almost the same activity as the wild-type CthIspH–RPS1 which contains 4 S1-like repeats, indicating that the RPS1 domain is not essential for IspH catalysis, at least in this *in vitro* assay.

**Fig. 5:**
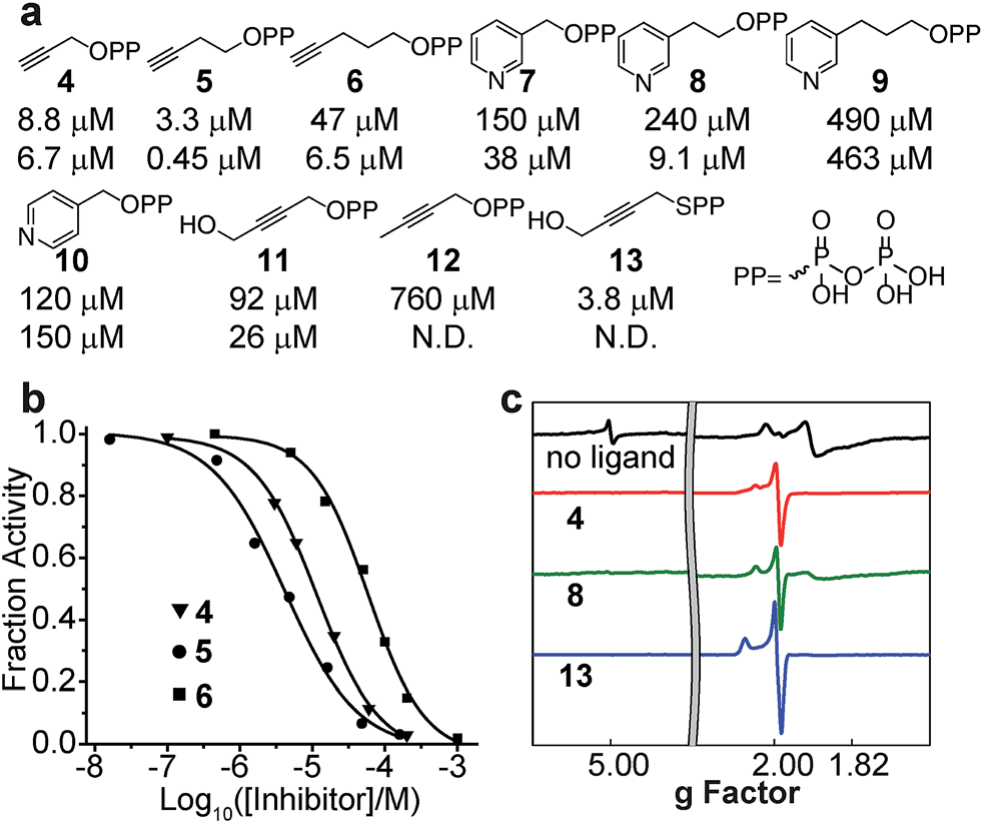
Inhibition of CthIspH–RPS1. (a) Compounds tested in the CthIspH–RPS1 inhibition assays and their IC_50_ values against CthIspH–RPS1 (top) and AaIspH (bottom). (b) Dose–response inhibition curves of three acetylene inhibitors. (c) 9 GHz EPR spectra of CthIspH–RPS1 reduced without ligand, reduced +**4**, reduced +**8**, and reduced +**13**.

We then investigated the 9 GHz EPR spectra of wild-type and truncation mutants of CthIspH–RPS1, Fig. 4f, to see if there were any obvious differences in cluster electronic structure, due to RPS1 binding. The wild-type CthIspH–RPS1 spectrum (Fig. 4f, pink) had *g* values of [2.022, 1.910, 1.896, 1.826] in the *g* ∼ 2 region, typical of reduced *S* = 1/2 Fe_4_S_4_ cluster. In addition, an isotropic signal at *g* = 5.0 was assigned to an *S* = 7/2 species (see Fig. S4† for a simulation), similar to a previously observed [Fe_4_S_4_]^+^ cluster from benzoyl-CoA reductase.^50^ Higher-spin species were not as obvious in *E. coli* IspH (Fig. 4f, yellow), and the *g* values at *g* ∼ 2 were also slightly lower [1.998, 1.900, 1.873, 1.791]. The mutants all exhibited essentially the same EPR spectra as the wild-type *C. thermocellum* protein. We also made (though not by design) a very short *C. thermocellum* IspH (residues 1-171; “Cth171”) in which the *C*-terminal “leaf” of the cloverleaf was excised. Although inactive and missing the third essential Cys, the protein still bound a reducible Fe/S cluster and had an EPR spectrum very similar to that of the other IspHs, as shown in Fig. 4g. However, unlike the other unliganded IspHs, Cth171 exhibited just the strong signal in the *g* ∼ 2 region, and the spectra did not change upon ligand (**3**, **4**, **5** and **8**) binding, Fig. 4g and 5. Apparently, the 3 Cys residues are not essential for cluster binding, but are for ligand binding.

### IspH–RPS1 inhibition and a comparison with other IspHs

We next investigated the inhibition of CthIspH–RPS1 catalytic activity with the inhibitors (**4–12**) developed previously,^7,30^ as well as a new compound (**13**). The interest here is that it might be possible to develop compounds that specifically inhibit *F. nucleatum* cell growth, reinstating, perhaps, the cytotoxicity of NK cells against colorectal carcinomas.^35–37^

Structures of the compounds tested are shown in Fig. 5a and consist of various alkyne and pyridine diphosphates and phosphonates. Representative dose–response inhibition curves are shown in Fig. 5b. These and other inhibition results are summarized in Fig. 5a together with *Aquifex aeolicus* IspH inhibition results. As a class, the acetylenes were the most potent inhibitors. The best inhibitor for CthIspH–RPS1 was the alkynyl compound **5**, which had an IC_50_ of 3.3 μM. Compound **5** was also the most potent alkyne inhibitor for AaIspH, where it had the lowest IC_50_, 0.45 μM. The pyridine-containing compounds were, in general, weak inhibitors. Interestingly, a novel alkynyl thioester compound (*i.e.* with a P–S bond), **13**, was also very active against CthIspH–RPS1, compared to the ester compound **11**. This thioester feature could potentially be useful in developing more drug-like leads that are resistant to hydrolysis, although pro-drugs will likely be needed for good cell penetration.

To investigate IspH–RPS1 ligand–protein interactions in more detail, we obtained 9 GHz EPR spectra of liganded (the acetylene **4**, the pyridine **8**, and the thiolo-diphosphate **13**), reduced CthIspH–RPS1, Fig. 5c. Upon binding of the alkynes **4** or **13**, the signal from the higher-spin species in the unliganded spectrum disappeared and the signal intensity of the spin *S* = 1/2 species increased, with *g* values of [2.099, 2.012, 1.999] in the case of **13**, similar to the values found with the alkyne inhibitors reported previously, bound to EcIspH and AaIspH.^7^ Pyridine compounds such as **7–10** are another class of IspH inhibitors, but these all had only weak activity against CthIspH–RPS1, resulting in a significant amount of unliganded signal in the EPR spectrum of **8**, despite the ligand being present in a 20-fold excess.

### IspH–RPS1 binds to *E. coli* transcription termination factor Rho

A surprising observation during protein expression and purification was that when heterologously expressed in *E. coli*, CthIspH–RPS1 always co-purified with a protein of 46 kDa, as seen in the MALDI spectrum in Fig. 4a. This protein (M_2_, Fig. 4a) bound strongly enough to CthIspH–RPS1 that it co-purified with a CthIspH–RPS1 construct containing both a His-Tag and a Strep-Tag, in a two-step affinity chromatographic purification. The binding partner was subsequently found to be the *E. coli* transcription termination factor Rho through protein MS fingerprint analysis (Fig. S5a†), with a sequence coverage ratio of 69%. The Rho factor is a helicase that unwinds the DNA–RNA junction during transcription termination and has a strong binding affinity for nucleotides.^51,52^ It seemed possible that the binding between CthIspH–RPS1 and EcRho might also be mediated by nucleotide binding and indeed, we found that Rho could be dissociated from CthIspH–RPS1 by washing the Ni-NTA resin with 0.5 mM of a (CT)_4_ DNA oligomer, a reported Rho binder in an X-ray study^53^ (Fig. S5b†). It is also of interest that there is an actual (*i.e.*, annotated) IspH–helicase fusion protein, in *Bacteroidales bacterium* CF, Fig. 2b.

### Possible roles of IspH–RPS1 and IspH–UbiA

**Fig. 6:**
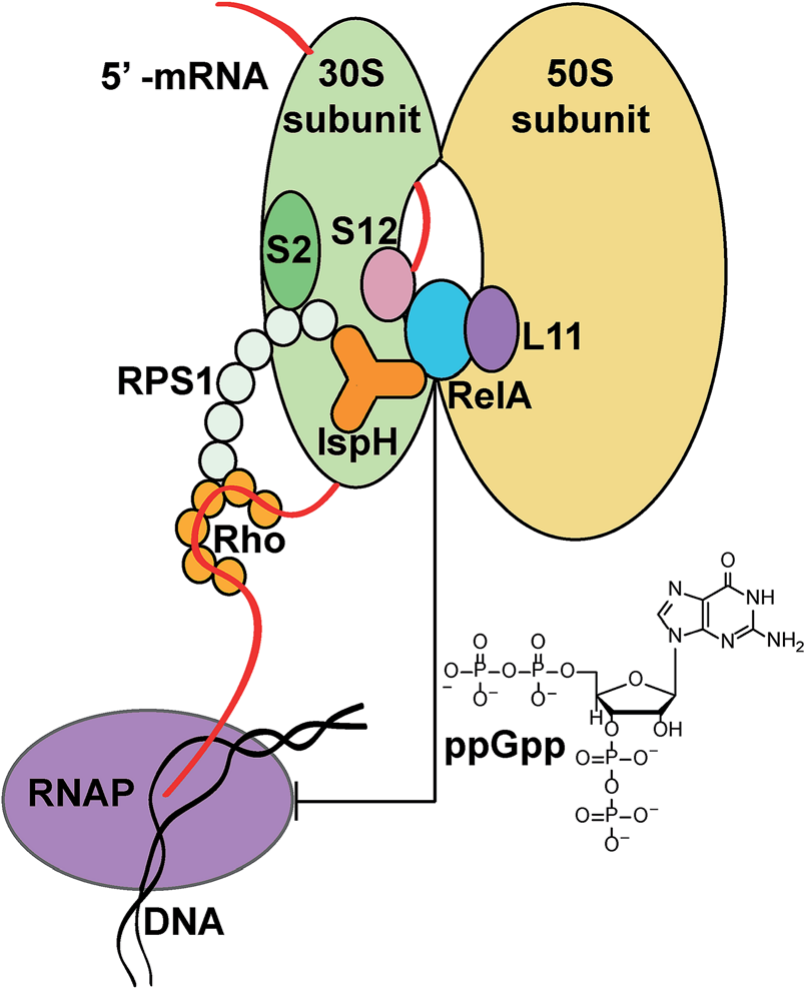
Possible IspH–RPS1/RelA/Rho ribosome interactions. Upon binding to the ribosome through the RPS1 domain, IspH could interact with RelA and affect its activity. The ppGpp released modulates a series of cell activities, for example inhibiting RNA polymerase (RNAP). The transcription termination factor Rho could also be affected upon interacting with IspH–RPS1 (as suggested from the MALDI-TOF experiments).

The roles of the IspH fusions in IspH–RPS1 and IspH–UbiA remain to be determined. What is, however, clearly of interest is that there are hundreds of anaerobes that contain IspH–RPS1 hybrids, many are found in the human gut, and some are pathogens or carcinogens. The very observation that IspH–RPS1 proteins exist supports previous ideas derived from work with *E. coli* (that lacks the RPS1 fusion) that the IspH domain might bind close to RelA when IspH–RPS1 binds to the ribosome, Fig. 6. Combining these results and ideas with the results of MS, EM and microbiological studies,^17–19,42^ we hypothesize that the 4Fe–4S cluster might act as a “switch” to probe iron or oxygen levels, affecting RelA activity and the stringent response, a moonlighting^54^ 4Fe–4S cluster containing protein that would be reminiscent of the fumarate–nitrate reductase regulatory protein, FNR,^55^ or perhaps of aconitase, another 4Fe–4S protein that has recently been found as a fusion with another ribosomal protein, bL21.^56^ While speculative, it seems possible that the 4^th^ water-ligated Fe ^13^ of the 4Fe–4S cluster may dissociate to trigger a conformational change of the protein, basically as found in early IspH structures with 3Fe–4S clusters.^8,57^

With IspH–UbiA, there are fewer species (47) than found with the IspH–RPS1 hybrids (447) but this is still a large number (*versus* that expected for random fusions,^24^ <1). Again, all are found in anaerobes, so IspH might (again) act either as a sensor, or, perhaps more likely, the UbiA domain might localize IspH to the cell membrane, with IspH facilitating UbiA-like-protein product formation by providing high local levels of IPP/DMAPP (and hence, down-stream prenyl diphosphates). We attempted expression of IspH–UbiA fusion proteins from several sulfate-reducing bacteria: *Desulfobacterium autotrophicum*, *Desulfobacca acetoxidans* and *Thermodesulfatator altanticus*, but only succeeded in producing modest levels of IspH together with a UbiA domain fragment, most protein being truncated and appearing in inclusion bodies. The observation of several other, single-example, fusion hybrids, Fig. S2,† could be the result of random events, however, it is of interest that in every case, the IspH partner is involved in phosphorus metabolism: a helicase, kinase, phosphatase or a phosphorylase.

## Conclusions

Overall, the results reported here are of interest since they show that active IspH–RPS1 proteins can be expressed, and inhibited, of potential interest in the context of understanding the biology underlying IspH gene fusions, as well as in anti-infective and even anti-cancer drug discovery. There are many hundreds of organisms containing IspH–RPS1, about 37% being human gut bacteria. Some of these are in pathogens such as *Clostridium tetani*, *Clostridium botulinum*, and in *Fusobacterium nucleatum*, an oral pathogen that is also being linked to colon carcinoma. We also found that there are ∼47 IspH–UbiA hybrids, all in sulfate-reducing bacteria, as well as smaller numbers of other IspH hybrids. We cloned, expressed and purified active IspH–RPS1 from *Clostridium thermocellum* and found that it had similar activity to IspH from another thermophile, *Aquifex aeolicus*. The IspH–RPS1 protein contains IspH fused to 4 RPS1 repeats: removal of 1, 2, 3, or 4 of these domains had no effect on IspH catalytic activity. We investigated the inhibition of *C. thermocellum* IspH catalytic activity (conversion of HMBPP to DMAPP and IPP) by a series of alkyne and pyridine diphosphate and related inhibitors finding most potent activity with a novel alkyne thiolo-diphosphate, and the profiles of activity against both *C. thermocellum* and *A. aeolicus* were very similar. We also found that IspH–RPS1 bound to an *E. coli* protein, Rho, a helicase, and that there was evidence for an IspH–helicase fusion hybrid, in *Bacteroidales bacterium* CF. With IspH–UbiA, we were not successful in expressing full length active protein, but the observation of such hybrids is still intriguing given that both components are likely to be involved in isoprenoid biosynthesis. The IspH–UbiA hybrid is very likely to fit the functional definition of a Rosetta stone protein in which there are two domains that act in the same metabolic pathway. IspH makes DMAPP and IPP, and UbiA-like proteins utilize a diverse range of prenyl diphosphates. With IspH–RPS1, there are many fusion hybrids reported, but the functional-relatedness of the two domains is less clear. However, since RPS1 proteins bind close to the RelA binding site on the bacterial ribosome, a role for IspH–RPS1 in the bacterial stringent-response is a possibility, consistent with previous suggestions of an IspH–RelA interaction, although further work will be required in order to fully understand the mechanism of action of both hybrids.
