# Supplementary material for: IspH–RPS1 and IspH–UbiA: “Rosetta stone” proteins
Source: Chem Sci. 2015 Sep 7;6(12):6813–22. doi: 10.1039/c5sc02600h (PMC4746011; doi:10.1039/c5sc02600h)
Supplement: Supplementary file 1 [file SC-006-C5SC02600H-s001.pdf]

Electronic Supplementary Material (ESI) for Chemical Science.
This journal is © The Royal Society of Chemistry 2015

Electronic Supplementary Information

IspH-RPS1 and IspH-UbiA: "Rosetta Stone" Proteins

Guodong Rao[a], Bing O'Dowd[a], Jikun Li[a], Ke Wang[a], and Eric Oldfield[a]*

a. Department of Chemistry, 600 S Mathews Avenue, University of Illinois at Urbana−Champaign, Urbana, IL, 61801
*Corresponding author: eoldfiel@illinois.edu Tel 217-333-3374, Fax 217-444-0997

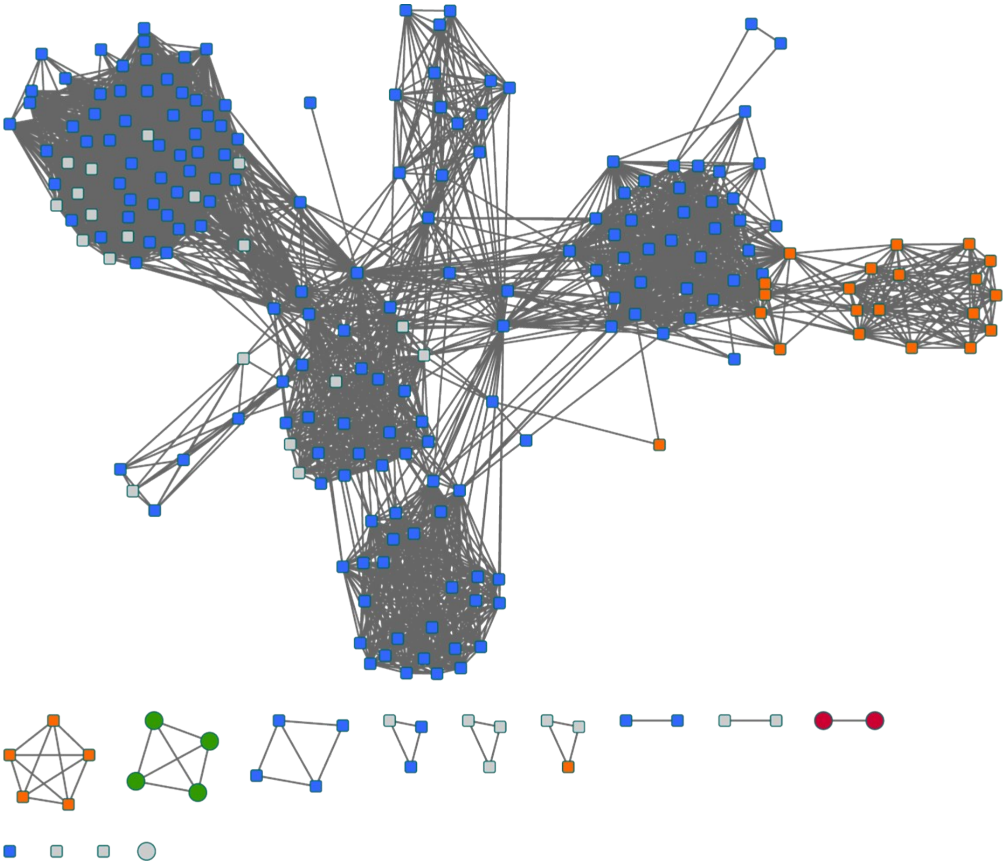

Figure S1. Sequence similarity network of IspH-RPS1 cluster, expectation-value (e-value) of 10^{-160}. Blue: Clostridia. Orange: Negativicutes. Green: Thermotogae. Red: Fusobacteria. Circle: IspH-RPS1 with 6 S1 repeats.

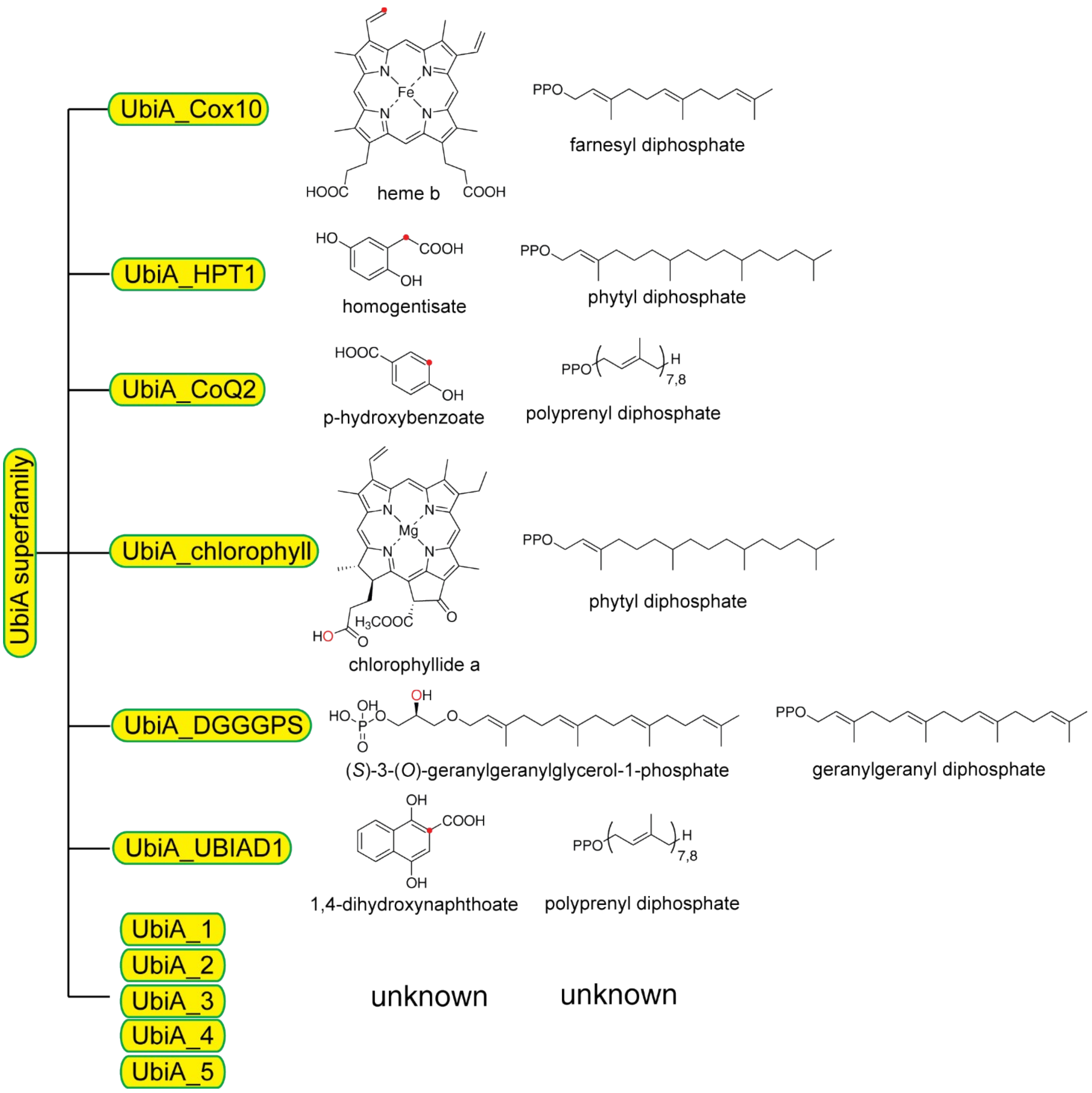

Figure S2. The UbiA superfamily. The UbiA superfamily has a very diverse range of substrates for prenylation and the substrates/products in several cases are not known. The sub-groups of UbiA and their substrates are listed. Atoms in red indicate the sites of prenylation. The UbiA in the IspH-UbiA hybrid belongs to the UbiA_5 category with unknown function.

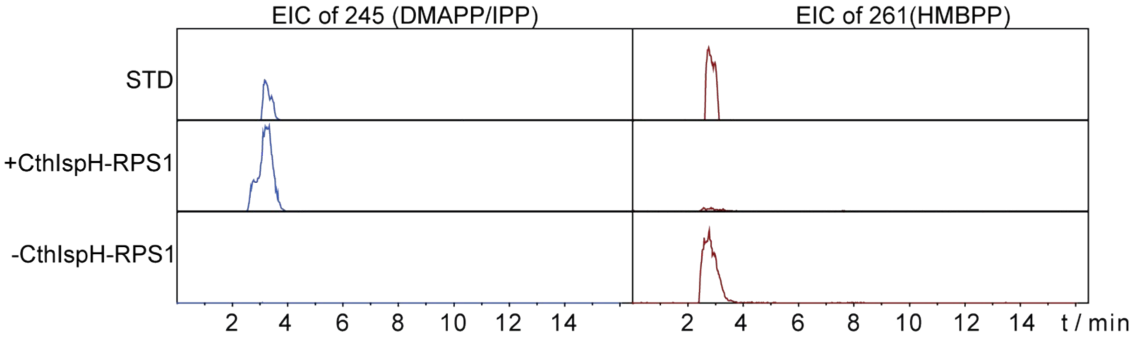

Figure S3. LC-MS of CthIspH-RPS1 catalyzed reaction confirmed the production of DMAPP/IPP and consumption of HMBPP. DMAPP and IPP were not separated in this chromatogram.

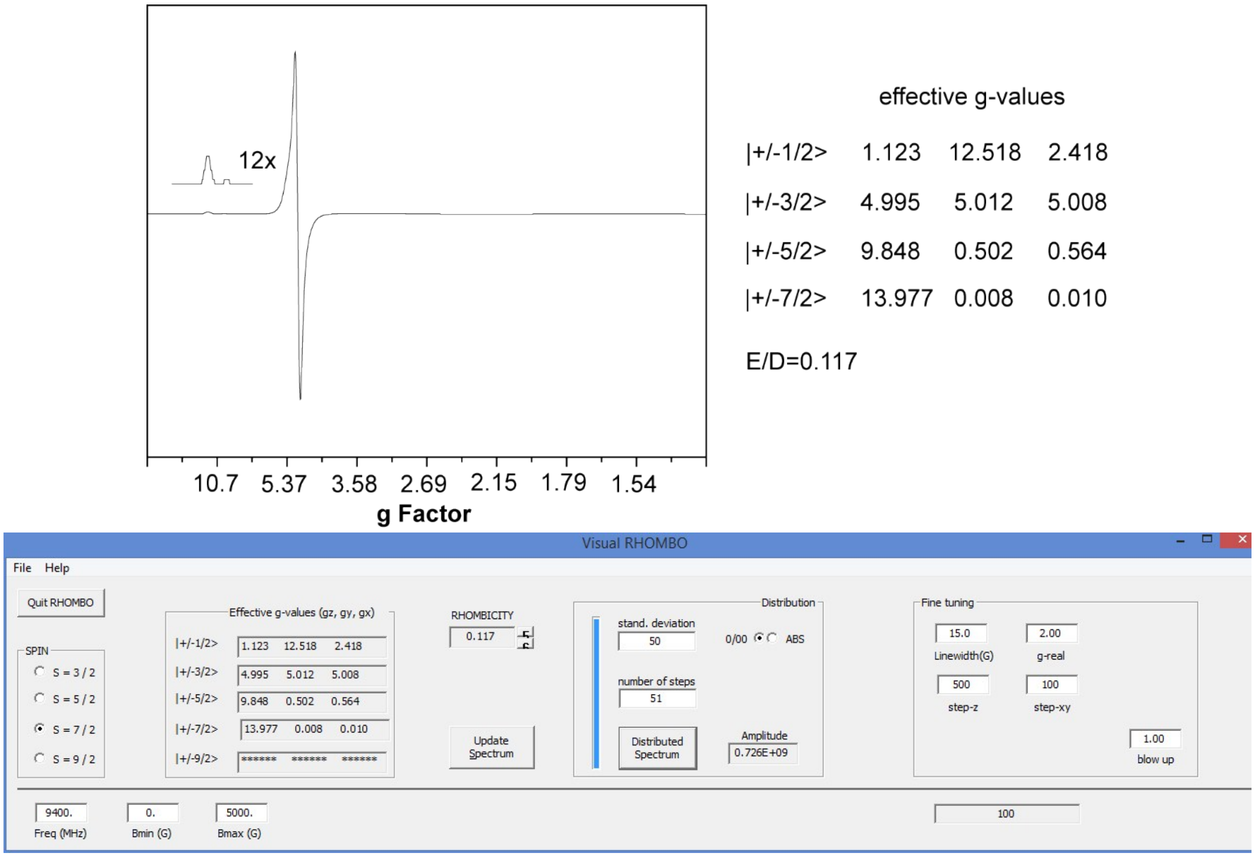

Figure S4. Simulation of the g=5.0 signal from the EPR spectrum of reduced CthIspH-RPS1 with an S=7/2 spin system and E/D=0.117. The total effective g-values are listed. Simulation was carried out using Visual RHOMBO (http://www.tnw.tudelft.nl/nl/over-faculteit/afdelingen/biotechnology/data-software/visual-rhombo/)

A

1 **MNLTELKNTP** **VSELITLGEN** **MGLENLAR**MR **KQDIIFAILK** QHAK**SGEDIF**
51 **GDGVLEILQD** **GFGFLRSADS** **SYLAGPDDIY** **VSLSQIRR**FN LRTGDTISGK
101 IRPPKEGERY FALLKVNEVN FDKPENARNK **ILFENLTPLH** **ANSR**LRMER**G**
151 **NGSTEDLTAR** **VLDLASPIGR** GQR**GLIVAPP** **KAGK**TMLLQN IAQSIAYNHP
201 DCVLMVLLID ERPEEVTEMQ R**LVKGEVVAS** **TFDEPASRHV** **QVAEMVIEK**A
251 KRLVEHKK**DV** **IILLDSITR**L AR**AYNTVVPA** **SGKVLTGGVD** **ANALHRPK**RF
301 FGAAR**NVEEG** **GSLTIIATAL** **IDTGSKMDEV** **IYEEFKGTGN** **MELHLSR**KIA
351 EK**RVFPAIDY** **NRSGTRKEEL** **LTTQEELQK**M WILR**KIIHPM** **GEIDAMEFLI**
401 **NK**LAMTK**TND** **DFFEMMKR**S

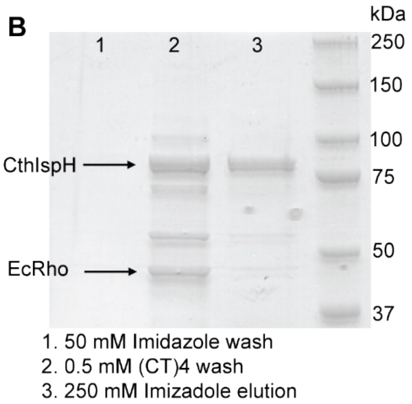

Figure S5. (A) *E. coli* Rho transcription termination factor MS fingerprint. Covered peptide fragments are shown in red. (B) SDS-PAGE gel showing that EcRho can be dissociated from IspH-RPS1 with a $(CT)_4$ DNA oligomer.
